# Supplementary material for: Microbiological Characterization of Protected Designation of Origin Serra da Estrela Cheese
Source: Foods. 2023 May 16;12(10):2008. doi: 10.3390/foods12102008 (PMC10217187; doi:10.3390/foods12102008)
Supplement: Supplementary file 1 [file foods-12-02008-s001.zip › foods-2339227-supplementary/Table S1.pdf]

	Milk	Cardoon			Curd			Cheese		
	(n=1)	Sample 1	Sample 2	Sample 3	Sample 1	Sample 2	Sample 3	Sample 1	Sample 2	Sample 3
Escherichia coli acc. ISO 16649-2:2001	n.d.	3.1 (0.2)	n.d.	n.d.	1.6 (0.2)	1.00 (0.00)	1.3 (0.4)	2.91 (0.04)	3.1 (0.1)	2.5 (0.4)
Enterobacteriaceae acc. 21528-2:2017	2.1 (0.1)	6.35 (0.00)	4.72 (0.05)	5.78 (0.00)	3.37 (0.08)	2.84 (0.02)	2.6 (0.2)	5.39 (0.05)	4.9 (0.2)	5.16 (0.08)
Bacillus cereus acc. ISO 7932:2004	n.d.	n.d.	1.6 (0.2)	1.64 (0.00)	n.d.	n.d.	n.d.	n.d.	n.d.	n.d.
**Coagulase-positive staphylococci** acc. ISO 6888-1:1999	n.d.	n.d.	n.d.	n.d.	3.69 (0.03)	4.18 (0.00)	3.73 (0.02)	n.d.	n.d.	n.d.
Listeria monocytogenes acc. 11290-2:2017	n.d.	n.d.	n.d.	n.d.	n.d.	n.d.	n.d.	n.d.	n.d.	n.d.
***Listeria* spp.** acc. 11290-2:2017	3.1 (0.1)	3.1 (0.1)	n.d.	n.d.	2.00 (0.08)	2.0 (0.1)	1.88 (0.07)	0.5 (0.7)	1.94 (0.04)	n.d.
Clostridium perfringens acc. ISO 7937:2004	ND	ND	ND	ND	ND	ND	ND	0.00 (0.00)	-0.01 (0.00)	-0.01 (0.00)
***Salmonella* spp.** acc. ISO 6579-1:2017	Absent (in 25g)	ND	ND	ND	Absent (in 25g)	Absent (in 25g)	Absent (in 25g)	Absent (in 25g)	Absent (in 25g)	Absent (in 25g)
**Yeasts** acc. ISO 21527-1:2008	3.6 (0.1)	6.16 (0.09)	5.16 (0.07)	4.8 (0.2)	3.85 (0.07)	3.9 (0.2)	3.9 (0.2)	2.8 (0.2)	2.95 (0.08)	2.8 (0.2)
**Moulds** acc. ISO 21527-1:2008	1.54 (0.09)	5.5 (0.2)	4.1 (0.3)	3.8 (0.4)	2.7 (0.4)	1 (1)	n.d.	2.3 (0.3)	2.1 (0.2)	2 (2)
**Psychrophiles**	ND	7.08 (0.00)	5.89 (0.06)	5.81 (0.08)	ND	ND	ND	5.60 (0.02)	5.4 (0.2)	5.82 (0.06)
Presumptive **Lactic Acid Bacteria** (total aerobic counts) on MRSA	5.17 (0.08)	n.d.	n.d.	n.d.	6.40 (0.09)	6.1 (0.2)	6.0 (0.2)	8.6 (0.2)	8.98 (0.05)	8.93 (0.07)
Presumptive **Lactic Acid Bacteria** (total anaerobic counts) on MRSA	5.2 (0.1)	3.1 (0.2)	n.d.	3.4 (0.3)	6.3 (0.1)	6.3 (0.3)	6.0 (0.1)	8.8 (0.2)	8.81 (0.07)	8.69 (0.06)
Presumptive **Lactococci** on M17	5.3 (0.2)	5.4 (0.2)	3.5 (0.1)	4.5 (0.3)	6.33 (0.07)	6.42 (0.06)	6.01 (0.08)	9.1 (0.1)	7.9 (0.2)	8.73 (0.04)
Presumptive **Lactobacilli** on RA	4.9 (0.2)	n.d.	n.d.	3.58 (0.09)	6.3 (0.1)	5.8 (0.1)	5.80 (0.08)	8.55 (0.08)	8.9 (0.1)	8.5 (0.2)
Presumptive **Enterococci** on SBA	4.4 (0.1)	3.9 (0.4)	n.d.	4.2 (0.1)	5.5 (0.1)	5.2 (0.2)	5.5 (0.1)	8.8 (0.2)	7.7 (0.2)	7.6 (0.1)
Presumptive ***Leuconostoc* spp.** on MSE	4.72 (0.04)	5.1 (0.1)	3.3 (0.7)	4.61 (0.07)	4.8 (0.1)	5.4 (0.4)	5.5 (0.2)	8.4 (0.1)	8.58 (0.06)	8.35 (0.05)

ND – Not Determined; n.d. – Not Detected; n – number of samples analyzed
