# Supplementary material for: Microbiological Characterization of Protected Designation of Origin Serra da Estrela Cheese
Source: Foods. 2023 May 16;12(10):2008. doi: 10.3390/foods12102008 (PMC10217187; doi:10.3390/foods12102008)
Supplement: Supplementary file 1 [file foods-12-02008-s001.zip › foods-2339227-supplementary/Table S2.pdf]

	Milk	Cardoon			Curd			Cheese		
	(n=1)	Sample 1	Sample 2	Sample 3	Sample 1	Sample 2	Sample 3	Sample 1	Sample 2	Sample 3
Escherichia coli acc. ISO 16649-2:2001	0.00 (0.00)	n.d.	1.9 (0.2)	n.d.	1.00 (0.00)	n.d.	0.5 (0.7)	3.2 (0.1)	2.7 (0.1)	2.6 (0.1)
Enterobacteriaceae acc. 21528-2:2017	1.8 (0.1)	5.3 (0.1)	6.91 (0.00)	5.30 (0.06)	2.8 (0.3)	3.68 (0.08)	2.9 (0.1)	5.05 (0.07)	4.99 (0.08)	4.05 (0.00)
Bacillus cereus acc. ISO 7932:2004	n.d.	1.8 (0.2)	1.47 (0.07)	1.3 (0.2)	n.d.	n.d.	n.d.	n.d.	n.d.	n.d.
**Coagulase-positive staphylococci** acc. ISO 6888-1:1999	n.d.	n.d.	n.d.	n.d.	n.d.	n.d.	n.d.	n.d.	n.d.	n.d.
Listeria monocytogenes acc. 11290-2:2017	n.d.	n.d.	n.d.	n.d.	n.d.	n.d.	n.d.	n.d.	n.d.	n.d.
***Listeria* spp.** acc. 11290-2:2017	n.d.	n.d.	2.9 (0.3)	n.d.	1.4 (0.5)	n.d.	n.d.	n.d.	n.d.	1.8 (0.2)
Clostridium perfringens acc. ISO 7937:2004	ND	ND	ND	ND	ND	ND	ND	n.d.	n.d.	n.d.
***Salmonella* spp.** acc. ISO 6579-1:2017	Absent (in 25g)	ND	ND	ND	Absent (in 25g)	Absent (in 25g)	Absent (in 25g)	Absent (in 25g)	Absent (in 25g)	Absent (in 25g)
**Yeasts** acc. ISO 21527-1:2008	4.4 (0.3)	4.99 (0.00)	5.6 (0.4)	4.0 (0.1)	4.3 (0.2)	4.0 (0.2)	3.5 (0.4)	3.0 (0.3)	3.40 (0.08)	2.7 (0.3)
**Moulds** acc. ISO 21527-1:2008	2 (2)	6.31 (0.09)	5.4 (0.1)	4.2 (0.1)	2 (2)	2 (2)	3.18 (0.00)	2.2 (0.3)	2.6 (0.2)	1 (2)
**Psychrophiles**	7.4 (0.3)	6.42 (0.03)	7.66 (0.09)	6.15 (0.09)	7.50 (0.03)	7.20 (0.08)	6.8 (0.2)	ND	ND	ND
Presumptive **Lactic Acid Bacteria** (total aerobic counts) on MRSA	6.3 (0.1)	n.d.	n.d.	3.1 (0.2)	7.0 (0.2)	6.7 (0.2)	6.19 (0.08)	9.0 (0.1)	8.9 (0.1)	8.9 (0.1)
Presumptive **Lactic Acid Bacteria** (total anaerobic counts) on MRSA	6.0 (0.2)	4.8 (0.1)	5.77 (0.09)	4.5 (0.2)	6.9 (0.2)	6.5 (0.2)	6.2 (0.1)	9.0 (0.3)	8.9 (0.1)	9.09 (0.05)
Presumptive **Lactococci** on M17	6.35 (0.02)	5.2 (0.1)	6.13 (0.08)	5.84 (0.01)	7.13 (0.06)	6.74 (0.07)	6.3 (0.1)	9.37 (0.03)	9.06 (0.09)	9.2 (0.2)
Presumptive **Lactobacilli** on RA	6.10 (0.01)	n.d.	5.84 (0.06)	3.5 (0.2)	6.95 (0.01)	6.3 (0.5)	6.0 (0.2)	9.12 (0.03)	8.81 (0.06)	8.9 (0.3)
Presumptive **Enterococci** on SBA	5.75 (0.01)	n.d.	5.8 (0.1)	4.0 (0.2)	6.32 (0.08)	5.97 (0.03)	5.8 (0.3)	8.0 (0.1)	7.96 (0.06)	7.84 (0.06)
Presumptive ***Leuconostoc* spp.** on MSE	5.7 (0.1)	n.d.	5.9 (0.1)	3.6 (0.4)	6.67 (0.08)	5.62 (0.06)	5.6 (0.2)	8.62 (0.07)	8.5 (0.1)	8.40 (0.02)

ND – Not Determined; n.d. – Not Detected
