# Supplementary material for: Microbiological Characterization of Protected Designation of Origin Serra da Estrela Cheese
Source: Foods. 2023 May 16;12(10):2008. doi: 10.3390/foods12102008 (PMC10217187; doi:10.3390/foods12102008)
Supplement: Supplementary file 1 [file foods-12-02008-s001.zip › foods-2339227-supplementary/Table S3.pdf]

| | Milk (n=1) | Cardoon (n=1) | Curd Sample 1 | Curd Sample 2 | Curd Sample 3 | Cheese Sample 1 | Cheese Sample 2 | Cheese Sample 3 |
|---|---|---|---|---|---|---|---|---|
| ***Escherichia coli*** acc. ISO 16649-2:2001 | n.d. | n.d. | n.d. | 1.4 (0.5) | 0.7 (0.9) | 2.4 (0.3) | 2.6 (0.2) | 2.2 (0.1) |
| ***Enterobacteriaceae*** acc. 21528-2:2017 | 1.2 (0.2) | 3.3 (0.1) | 2.38 (0.08) | 2.56 (0.06) | 2.37 (0.08) | 3.96 (0.02) | 3.80 (0.03) | 4.14 (0.06) |
| ***Bacillus cereus*** acc. ISO 7932:2004 | n.d. | n.d. | n.d. | n.d. | n.d. | n.d. | n.d. | n.d. |
| **Coagulase-positive staphylococci** acc. ISO 6888-1:1999 | n.d. | n.d. | n.d. | n.d. | n.d. | n.d. | n.d. | n.d. |
| ***Listeria monocytogenes*** acc. 11290-2:2017 | n.d. | n.d. | n.d. | n.d. | n.d. | n.d. | n.d. | n.d. |
| ***Listeria* spp.** acc. 11290-2:2017 | n.d. | n.d. | n.d. | 1.1 (0.4) | 0.70 (0.00) | 1.8 (0.2) | n.d. | n.d. |
| ***Clostridium perfringens*** acc. ISO 7937:2004 | ND | ND | ND | ND | ND | n.d. | n.d. | 0.05 (0.00) |
| ***Salmonella* spp.** acc. ISO 6579-1:2017 | Absent (in 25g) | ND | Absent (in 25g) | Absent (in 25g) | Absent (in 25g) | Absent (in 25g) | Absent (in 25g) | Absent (in 25g) |
| **Yeasts** acc. ISO 21527-1:2008 | 3.30 (0.06) | 3.32 (0.08) | 3.35 (0.06) | 3.76 (0.06) | 3.6 (0.3) | 3.7 (0.7) | 3.9 (0.2) | 3.7 (0.2) |
| **Moulds** acc. ISO 21527-1:2008 | 3.1 (0.2) | 3.6 (0.1) | 3.3 (0.3) | 3.87 (0.09) | 3.3 (0.3) | 3.00 (0.00) | 3.1 (0.2) | 2.9 (0.3) |
| **Psychrophiles** | 4.96 (0.00) | 5.7 (0.1) | 5.00 (0.03) | 5.20 (0.2) | 5.1 (0.4) | 5.8 (0.3) | 6.0 (0.2) | 5.8 (0.1) |
| Presumptive **Lactic Acid Bacteria** (total aerobic counts) on MRSA | 5.4 (0.1) | 3.0 (0.1) | 6.59 (0.07) | 7.06 (0.02) | 7.2 (0.2) | 8.95 (0.04) | 8.79 (0.03) | 8.74 (0.04) |
| Presumptive **Lactic Acid Bacteria** (total anaerobic counts) on MRSA | 5.26 (0.05) | 3.1 (0.2) | 6.5 (0.2) | 7.09 (0.04) | 7.3 (0.3) | 8.95 (0.02) | 8.85 (0.03) | 8.82 (0.03) |
| Presumptive **Lactococci** on M17 | 5.4 (0.3) | 5.07 (0.04) | 6.8 (0.3) | 7.40 (0.01) | 7.45 (0.04) | 9.3 (0.1) | 9.3 (0.1) | 9.21 (0.08) |
| Presumptive **Lactobacilli** on RA | 5.3 (0.2) | 3.0 (0.3) | 7.0 (0.1) | 7.5 (0.1) | 7.33 (0.07) | 9.1 (0.1) | 8.9 (0.1) | 8.97 (0.08) |
| Presumptive **Enterococci** on SBA | 4.5 (0.2) | 3.65 (0.07) | 5.6 (0.4) | 5.9 (0.1) | 5.88 (0.09) | 7.1 (0.2) | 7.2 (0.1) | 7.2 (0.1) |
| Presumptive ***Leuconostoc* spp.** on MSE | 5.0 (0.3) | 3.6 (0.3) | 6.3 (0.2) | 6.4 (0.3) | 6.46 (0.02) | 8.81 (0.09) | 8.7 (0.3) | 8.3 (0.2) |

ND – Not Determined; n.d. – Not Detected
